# Habitual Preference for the Nondrug Reward in a Drug Choice Setting

**DOI:** 10.3389/fnbeh.2020.00078

**Published:** 2020-05-25

**Authors:** Youna Vandaele, Karine Guillem, Serge H. Ahmed

**Affiliations:** ^1^Department of Psychiatry, Center for Psychiatric Neuroscience, Lausanne University Hospital, Lausanne, Switzerland; ^2^Institut des Maladies Neurodégénératives, UMR 5293, Université de Bordeaux, Bordeaux, France; ^3^CNRS, Institut des Maladies Neurodégénératives, UMR 5293, Bordeaux, France

**Keywords:** choice, cocaine, saccharin, sweetness, habit, goal-directed

## Abstract

For adaptive and efficient decision making, it must be possible to select between habitual alternative courses of action. However, research in rodents suggests that, even in the context of simple decision-making, choice behavior remains goal-directed. In contrast, we recently found that during discrete trial choice between cocaine and water, water-restricted rats preferred water and this preference was habitual and inflexible (i.e., resistant to water devaluation by satiation). Here we sought to test the reproducibility and generality of this surprising finding by assessing habitual control of preference for saccharin over cocaine in non-restricted rats. Specifically, after the acquisition of preference for saccharin, saccharin was devalued and concurrent responding for both options was measured under extinction. As expected, rats responded more for saccharin than for cocaine during extinction, but this difference was unaffected by saccharin devaluation. Together with our previous research, this result indicates that preference for nondrug alternatives over cocaine is under habitual control, even under conditions that normally support goal-directed control of choice between nondrug options. The possible reasons for this difference are discussed.

## Introduction

Organisms are constantly choosing between alternatives to select appropriate actions based on prior experience or expected outcomes. Evidence indicates that the performance of reward-related actions in both rats and humans reflects the interaction of two learning processes (Dickinson and Balleine, 1994; Dickinson, 1994; Balleine and Dickinson, 1998). The deliberative goal-directed process depends on a representation of the outcome as a goal and requires encoding of both the outcome value and the instrumental contingency between the action and the outcome (Dickinson and Balleine, 1994; Balleine and Dickinson, 1998). In contrast, the habitual learning process dissociates actions from the evaluation of their consequences, such that habitual actions can be spontaneously elicited by particular situations or stimuli (Yin and Knowlton, 2006; Hart et al., 2014). The balance between goal-directed and habitual processes allows adaptive and efficient decision making. Although one may intuitively think that habitual course of actions can be selected among other alternatives, research in laboratory animals suggests that, even in the context of the simple choice decision, choice performance is dominated by goal-directed actions, rather than habitual responses (Colwill and Triola, 2002; Holland, 2004; Kosaki and Dickinson, 2010; Halbout et al., 2016). For instance, using the concurrent schedule in which two responses yielded different outcomes, post-training decrease in the incentive value of one outcome has been found to attenuate the rate of performance of the associated action, and to favor the choice of the alternative action (Yin et al., 2005; Corbit et al., 2013; Parkes and Balleine, 2013), indicating that choice behavior is goal-directed.

In a series of experiments, we have repeatedly shown that when facing a choice between pressing a lever to get a nondrug reward (i.e., water sweetened with saccharin) or an alternative lever to receive an intravenous dose of cocaine, most rats prefer the nondrug alternative (Lenoir et al., 2007; Cantin et al., 2010; Augier et al., 2012; Madsen and Ahmed, 2015; Vandaele et al., 2016). Importantly, we have found that choice could be biased in favor of cocaine by systematically varying the cost to obtain saccharin or by decreasing its concentration (Cantin et al., 2010). These results suggest that preference remains sensitive to instrumental and environmental contingencies, and may thus be under goal-directed control. However, we recently showed that this is, in fact, not the case (Vandaele et al., 2019b). Specifically, rats persisted to choose water, their preferred nondrug option when thirsty, even after devaluation by satiation and even if they consumed little of it upon delivery.

This result contrasts with the studies mentioned above showing that expression of habit is prevented in situations of choice involving multiple response-outcome associations (Colwill and Triola, 2002; Holland, 2004; Kosaki and Dickinson, 2010; Halbout et al., 2016). This discrepancy could be explained by the relative difference between the incentive value of the two outcomes which was large in our procedure (i.e., water under water-restriction vs. cocaine) but relatively small in prior studies (i.e., sucrose solution vs. sucrose pellets and sucrose solution vs. food pellets). In theory, when the options’ values are close, the comparison process is difficult and should thus engage goal-directed processes, whereas when outcomes’ values are sufficiently distant, a simple stimulus-response policy, relying on prior reward history, should suffice, eventually taking over goal-directed processes. Alternatively, this discrepancy could also be explained by other factors. First, the devalued outcome was water, a non-palatable biological reward that is essential for survival, particularly under conditions of water restriction (Vandaele et al., 2019b). Second, unlike prior studies, in our study, preference sensitivity to devaluation was not tested under extinction and with continuous access to both response options. Finally, in our study, devaluation also involved non-contingent access to the devalued outcome between choice trials, which may have resulted in concurrent degradation of instrumental contingency.

Here, we aimed at assessing habitual control of choice between a drug and a nondrug reward by using more standard devaluation procedures. Specifically, non-restricted rats were trained to choose between saccharin and cocaine. After the acquisition of preference for saccharin, saccharin was devalued and concurrent responding for both options was measured under extinction. Saccharin was devalued using two standard devaluation methods—sensory-specific satiety and conditioned taste aversion (CTA). As expected, rats responded more for saccharin than for cocaine during extinction, but this difference was unaffected by any method of saccharin devaluation. Together with our previous research, this result indicates that preference for nondrug alternatives over cocaine is under habitual control, even under conditions that normally support goal-directed control of choice between nondrug options close in value.

## Materials and Methods

### Subjects

Twenty male Sprague–Dawley rats (Charles River, L’Arbresle, France, 249-340 g at the beginning of experiments) were used. Rats were housed in groups of 2–3 and maintained in a temperature-controlled vivarium with a 12-h light-dark cycle. Food and water were freely available in the home cages and rats were neither food- nor water-restricted during behavioral testing. All experiments were carried out following institutional and international standards of care and use of laboratory animals UK Animals (Scientific Procedures) Act, 1986; and associated guidelines; the European Communities Council Directive (2010/63/UE, 22 September 2010) and the French Directives concerning the use of laboratory animals (décret 2013–118, 1 February 2013). The animal studies were reviewed and approved by the Committee of the Veterinary Services Gironde, agreement number B33-063-5.

### Apparatus

Twelve identical operant chambers (30 × 40 × 36 cm) were used for all behavioral training and testing (Imetronic, Pessac, France). These chambers have been described in detail elsewhere (Augier et al., 2012). Briefly, each chamber was equipped with two automatically retractable levers (Imetronic), a commercially-available lickometer circuit (Imetronic), two syringe pumps, a single-channel liquid swivel (Lomir biomedical Inc., Quebec, Canada) and two pairs of infrared beams to measure horizontal cage crossings.

### Surgery

Rats were surgically prepared with chronic Silastic catheters (Dow Corning Corporation, Michigan, MI, USA) in the right jugular vein that exited the skin in the middle of the back about 2 cm below the scapulae as described previously (Lenoir et al., 2013).

### Operant Training for Cocaine and Saccharin Self-administration

**Figure 1:**
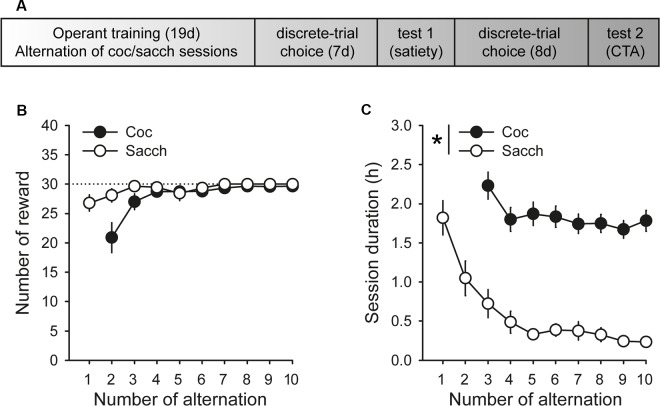
Timeline and operant training. **(A)** Timeline of experimental training and testing. **(B)** Mean (±SEM) number of cocaine (black circles) and saccharin (white circles) rewards self-administered across training sessions. The black dotted line indicates the maximum number of rewards allowed per session. **(C)** Mean duration (±SEM) of cocaine and saccharin sessions. The session duration variable was not properly saved during the first cocaine training session. *p < 0.0001 cocaine vs. saccharin.

Animals were first trained on alternate daily sessions to lever press for either water sweetened with saccharin (0.2% for 20 s, delivered in the drinking cup) or intravenous cocaine (0.25 mg delivered over 5 s) under a fixed-ratio 1 (FR1 time-out 20 s) schedule (i.e., one response results in one reward), as described in detail elsewhere (Lenoir et al., 2013). One lever was associated with cocaine reward (lever C), the other with saccharin reward (lever S). Sessions began with the extension of one single lever (C or S). If rats responded on the available lever, they were rewarded by the corresponding reward (cocaine or saccharin). Reward delivery was signaled by a 20-s illumination of the cue-light above the lever during which responses were not rewarded (i.e., time-out period). Sessions ended after rats had earned a maximum of 30 rewards or 3 h had elapsed. The maximum number of saccharin or cocaine rewards was limited to 30 per session to ensure approximately equal exposure to both rewards before choice testing. Importantly, to equate training conditions, rats were also tethered to the infusion line during saccharin training sessions but received no injections. There were a total of 10 saccharin training sessions that alternated with 9 cocaine training sessions (Figure 1A).

### Discrete-Trials Choice Procedure

After the acquisition of lever pressing for cocaine and saccharin, rats were allowed to choose during several consecutive daily sessions between the lever associated with cocaine (lever C) and the lever associated with saccharin (lever S) on a discrete-trials choice procedure. Each daily choice session consisted of 12 discrete trials, spaced by 10 min, and divided into two successive phases, sampling (four trials) and choice (eight trials). During sampling, each trial began with the presentation of one single lever in this alternative order: C—S—C—S. Lever C was presented first to prevent an eventual drug-induced taste aversion conditioning or negative affective contrast effects (Lenoir et al., 2007). If rats responded within 5 min on the available lever, they were rewarded by the corresponding reward (i.e., 0.25 mg cocaine delivered intravenously or 20-s access to water sweetened with 0.2% saccharin, as described above). Reward delivery was signaled by retraction of the lever and a 40-s illumination of the cue-light above this lever. If rats failed to respond within 5 min, the lever retracted and no cue-light or reward was delivered. Thus, during sampling, rats were allowed to separately evaluate each reward before making their choice. During choice, each trial began with the simultaneous presentation of both levers S and C. Rats had to select one of the two levers. During choice, reward delivery was signaled by retraction of both levers and a 40-s illumination of the cue-light above the selected lever. If rats failed to respond on either lever within 5 min, both levers retracted and no cue-light or reward was delivered. The response requirement of each reward was set to two consecutive responses to avoid eventual accidental choice. A response on the alternate lever before the satisfaction of the response requirement resets it. Response resetting occurred very rarely, however. Rats were tested in this discrete-trials choice procedure during at least five daily sessions until stabilization of group-average preference (i.e., no increasing or decreasing trend across three consecutive sessions and between-session variation <10%; Figure 1A).

### Satiety-Induced Saccharin Devaluation

Animals were divided into two groups, devalued (D) and non-devalued (ND). Animals were individually placed in feeding cages, brought to an experimental room (satiety room) physically different from the room containing the self-administration chambers (choice room), and allowed to acclimate to this room for 30 min. Rats in the D group (*N* = 10) were given 30 min of free access to a bottle containing 0.2% saccharin whereas rats in the ND group (*N* = 10) were given free access to a water bottle. The control solution was water because it has a distinct taste from saccharin but, like saccharin, is non-caloric. Immediately after home-cage pre-feeding, rats were brought to the choice room and tested for lever-press responding during extinction. For each rat, extinction begins after a delay of 10 min. During extinction, both levers are presented simultaneously and continuously for 10 min. Responding on either lever has no programmed consequence. To confirm the presence of saccharin satiety, immediately after extinction testing, animals were brought back to the satiety room and were all given free access to a bottle containing 0.2% saccharin during 30 min.

### Conditioned Taste Aversion (CTA)-Induced Saccharin Devaluation

Aversion conditioning was conducted in feeding cages in an experimental room (CTA room) physically different from the room containing the self-administration chambers (choice room) to minimize direct aversive conditioning to the operant chambers and to devalue saccharin in similar conditions as the satiety-induced devaluation test. Animals were allowed to acclimate to this room for 30 min to avoid novelty-induced anxiety. Rats in the D group (*N* = 10) were given 30 min of free access to a bottle containing 0.2% saccharin whereas rats in the ND group (*N* = 10) were given free access to a water bottle. After this 30-min period, rats returned to the colony room and were injected with lithium chloride (5 ml/kg, i.p., of 0.3 M LiCl) before being returned to their home cages. The entire procedure was repeated three times until >80% suppression of saccharin drinking. Rats were then left in their home cages for at least 48 h after the last LiCl administration and before being tested for lever-press responding under extinction. For each rat, extinction begins after a delay of 10 min. During extinction, both levers are presented simultaneously and continuously for 10 min. Responding on either lever has no programmed consequence. To confirm the presence of the CTA, immediately after extinction testing, animals were brought back to the CTA room and were all given free access to a bottle containing 0.2% saccharin during 30 min.

### Data Analysis

All data were subjected to mixed analyses of variance (ANOVA), followed by *post hoc* comparisons using Tukey’s Honestly Significant Difference (HSD) test. Comparisons with a fixed theoretical level (e.g., 50%) were conducted using one-sample *t*-tests. Some behavioral variables did not follow a normal distribution and were thus analyzed using non-parametric statistics (i.e., Friedman’s test for the main effect followed by Wilcoxon’s test for paired comparisons; Mann–Whitney for group comparison).

## Results

**Figure 2:**
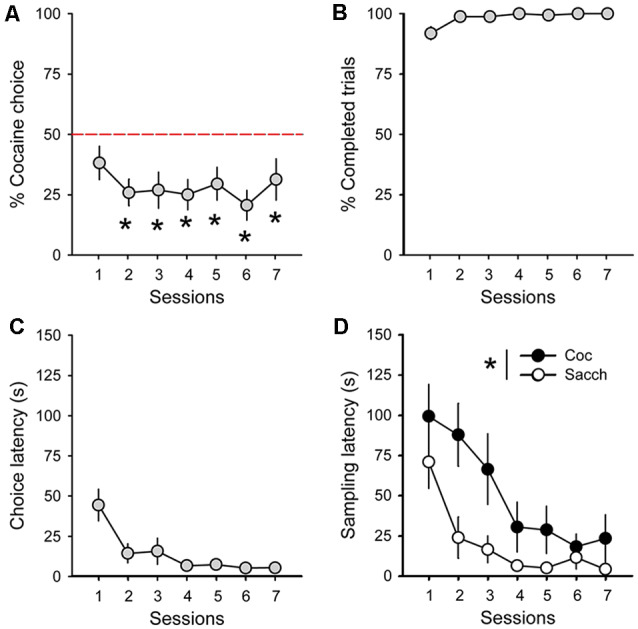
Rats prefer saccharin over cocaine. **(A)** Mean (±SEM) percentage of cocaine choice across sessions. The red dotted line represents the indifference level. *p < 0.05 against indifference. **(B)** Mean (±SEM) percentage of completed trials across choice sessions. **(C)** Mean (±SEM) choice latency across choice sessions. **(D)** Mean (±SEM) cocaine (black circles) and saccharin (white circles) sampling latency across choice sessions. *p < 0.05 compared to saccharin.

During acquisition, rats learned to self-administer saccharin and cocaine on alternate daily sessions and rapidly earned the maximum number of reward possible in both conditions (Figure 1B). However, rats self-administered saccharin at a much higher response rate than cocaine which resulted in shorter session durations (Figure 1C; main effect of reward: *F*_(1,18)_ = 75.91, *p* < 0.0001). Following this result and as expected from previous research, virtually all Sprague–Dawley rats preferred saccharin over cocaine when offered a choice (mean cocaine choice over the last three sessions: 27.1 ± 6.5%; Figure 2A). Their preference significantly deviated from indifference from the second session (*t*-values > 2.2, *p*-values < 0.05; Figure 2A). Although preference did not significantly change across sessions (Friedman ANOVA Chi Sqr = 7.2, *p* > 0.1), rats generally completed every choice trial (i.e., 99.8 ± 0.2%; Figure 2B) with increasing efficiency, as evidenced by the decrease in choice latency reaching about 5 s across the last three sessions (Friedman ANOVA Chi Sqr: 68.5, *p* < 0.0001; Figure 2C). This decrease in choice latency was accompanied with a decrease in both cocaine and saccharin sampling latency (cocaine: Friedman ANOVA Chi Sqr = 44.0, *p* < 0.0001; saccharin: Friedman ANOVA Chi Sqr = 46.6, *p* < 0.0001; Figure 2D) suggesting that rats learned to select options and to choose between them with little hesitation. Following rats’ preference, the latency to sample saccharin was shorter than the latency to sample cocaine (*F*_(1,19)_ = 10.40, *p*-values < 0.001; Figure 2D).

**Figure 3:**
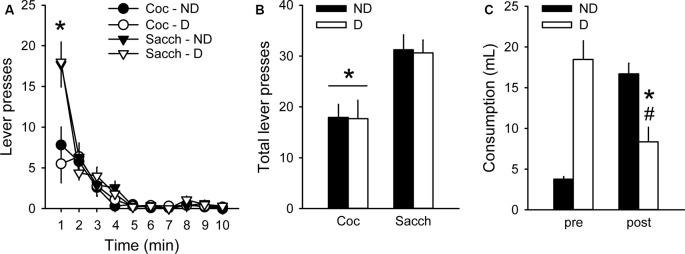
Choice performance is unaffected by the devaluation of saccharin with sensory-specific satiety. **(A)** Mean (±SEM) number of cocaine (circles) and saccharin (triangles) lever presses in the Non-Devalued (ND; black) and Devalued (D; white) groups, across 1-min time bins in the extinction test. *p < 0.01: Coc vs. Sacch. **(B)** Mean (±SEM) number of lever presses for cocaine and saccharin in the ND (black bars) and D (white bars) groups. *p < 0.001: Coc vs. Sacch. **(C)** Mean (±SEM) pre-test and post-test reward consumption in the ND (black bars) and D (white bars) groups. Rats in the ND group received water in the pre-test condition. *p < 0.01: post vs. pre. ^#^*p* < 0.01: D vs. ND.

We then assessed whether choice behavior in our procedure was sensitive to 30-min free access to either a bottle containing 0.2% saccharin (D group) or a bottle of water (ND group) before testing under extinction. Although animals having free access to saccharin drank a large amount of saccharin (18.1 ± 2.1 ml), this pre-feeding did not affect responding during the extinction test (group: *F*_(1,18)_ = 0.02, *p* > 0.5; Figures 3A,B). Animals in both groups responded more on the saccharin lever than on the cocaine lever (lever: *F*_(1,18)_ = 17.45, *p* < 0.001; Figures 3A,B), in agreement with their strong preference for saccharin. The greatest difference in responding between saccharin and cocaine occurred during the first minute of the test (time bin 1: *z* = 2.91, *p* < 0.01; Figure 3A). However, rats in the D group responded as much on the saccharin and cocaine lever as rats in the ND group (group × lever: *F*_(1,18)_ = 0.004, *p* > 0.5; Figures 3A,B). The lack of devaluation effect was not due to a failure of saccharin pre-feeding to induce sensory-specific satiety. Indeed, after the extinction test, animals in the D group significantly decreased their saccharin intake compared to their consumption during saccharin pre-feeding before the extinction test (*F*_(1,9)_ = 17.72, *p* < 0.01; Figure 3C). Furthermore, these rats consumed significantly less saccharin after the extinction test than rats in the ND group, previously exposed to water bottles (*F*_(1,18)_ = 13.84, *p* < 0.01; Figure 3C). Thus, although saccharin pre-feeding reliably induced sensory-specific satiety, animals were insensitive to reward devaluation suggesting that their behavior was under habitual control.

**Figure 4:**
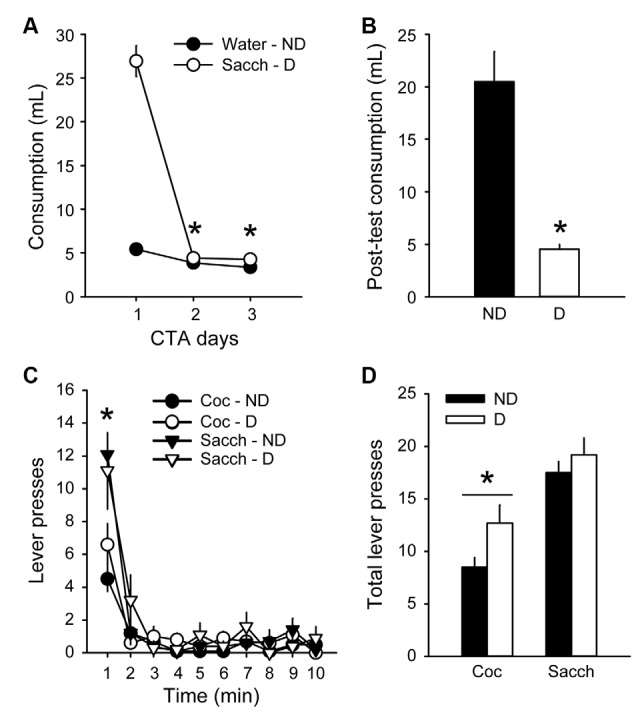
Choice performance is unaffected by the devaluation of saccharin with conditioned taste aversion (CTA). **(A)** Mean (±SEM) consumption of water or saccharin in the ND (black circles) and D (white circles) groups, respectively. *p < 0.001 compared to day 1. **(B)** Mean (±SEM) post-test consumption of saccharin in ND (black bar) and D (white bar) groups. *p < 0.0001 compared to ND. **(C)** Mean (±SEM) number of cocaine (circles) and saccharin (triangles) lever presses in the Non-Devalued (ND; black) and Devalued (D; white) groups, across 1-min time bins in the extinction test. *p < 0.01: Sacch vs. Coc. **(D)** Mean (±SEM) number of lever presses for cocaine and saccharin in the ND (black bars) and D (white bars) groups. *p < 0.0001: Coc vs. Sacch.

To further probe the resistance to devaluation, we assessed the effects of CTA on responding during extinction. Rats were first trained for 8 additional sessions in the discrete-trials choice procedure and maintained a stable preference for saccharin, similar to the preference before the first devaluation test (data not shown; average % of cocaine choice over the last three sessions: 26.8 ± 7.1). An aversion to saccharin was then conditioned by pairing its consumption with illness induced by lithium chloride (LiCl) for 3 days. LiCl injections induced a robust CTA (group × day: *F*_(2,36)_ = 117.4, *p* < 0.0001; Figures 4A,B) as animals in the D group decreased their saccharin intake between the first and the last day of LiCl treatment (from 26.9 ± 1.8 ml to 4.3 ± 0.2 ml; Tukey *p* < 0.001; Figure 4A). However, the LiCl devaluation had no effect on responding during the extinction test (group × lever: *F*_(1,18)_ = 0.65, *p* > 0.4; Figures 4C,D). Animals in both groups responded more on the saccharin lever than on the cocaine lever (*F*_(1,18)_ = 24.84, *p* < 0.0001), mainly during the first minute of the test (time × lever: *F*_(9,162)_ = 6.65, *p* < 0.0001; *post hoc* time bin 1; *p* < 0.0001; Figure 4C), in agreement with their preference and with the results described above (Figure 3A). The lack of devaluation effect was not due to a failure to induce CTA since animals in the D group significantly decreased their saccharin intake compared to the ND group during free access to saccharin bottle after the extinction test (Mann–Whitney, *Z* = 3.62, *p* < 0.0001; Figure 4B).

## Discussion

The present study clearly shows that choice between saccharin and cocaine is insensitive to changes in saccharin value, a hallmark of habitual performance (Balleine and Dickinson, 1998; Balleine and O’Doherty, 2010). As expected, rats responded more for saccharin than for cocaine during extinction, but this difference was unaffected by any method of saccharin devaluation (i.e., sensory-specific satiety or induction of a CTA). Together with our previous research (Vandaele et al., 2019b), this result indicates that preference for nondrug alternatives over cocaine is under habitual control, even under conditions that normally support goal-directed control of choice between nondrug options.

In our previous study, preference sensitivity to water devaluation was tested during reinforced choice trials, with free water accesses before and during the test, in conditions where drinking water constituted a biological need critical for survival (Vandaele et al., 2019b). These factors could have promoted preference insensitivity to devaluation. In the present study, several changes were done to avoid this potential caveat and to test sensitivity to outcome devaluation under more standard conditions (Holland, 2004; Kosaki and Dickinson, 2010; Corbit et al., 2013; Parkes and Balleine, 2013). Here, devaluation tests were conducted under extinction, with a 10 min concurrent access to both levers, allowing rats to freely sample them in a self-paced manner. Responding during both devaluation tests reflected rats’ preference with a higher rate of responding for saccharin compared to cocaine, specifically during the first minute of the test session. This result is in agreement with previous findings showing a stronger resistance to extinction on the saccharin lever (Cantin et al., 2010). However, we did not observe any effect of devaluation, indicating that choice performance was habitual. This finding is difficult to conciliate with previous empirical and theoretical research on choice behavior showing that training on a schedule offering a choice between responses yielding different outcomes prevents the expression of habits (Colwill and Rescorla, 1985; Holland, 2004; Kosaki and Dickinson, 2010). In two of the latter studies (Colwill and Rescorla, 1985; Kosaki and Dickinson, 2010), the CTA was conducted in the operant chambers whereas it was conducted in separate feeding cages in the present study. This was done on purpose to avoid any aversion conditioning to the choice context itself (Boakes et al., 1997; Kislal and Blizard, 2016, 2018). One could, therefore, argue that our negative findings may be due to a failure of generalization of the CTA to the choice context. This is unlikely, however, since such generalization has been previously observed in other similar studies (Dickinson et al., 1983; Holland, 2004; Schoenbaum and Setlow, 2005; Vandaele et al., 2017; Keiflin et al., 2019). Also, we have independent evidence that in our conditions, devaluation by satiation is effectively transportable across different contexts (in preparation). Taken together these considerations strongly suggest that saccharin choice is habitual in our choice procedure.

It is well known that overtraining on a particular response can render it habitual through the development of stimulus-response associations (Adams, 1982; Dickinson, 1985; Dickinson et al., 1995; Coutureau and Killcross, 2003). During initial operant training, animals were exposed to 300 saccharin outcomes, a number of trials sufficient to shift animals’ performance from goal-directed to habitual (Dickinson et al., 1995). It could then be argued that repeated testing could account for the insensitivity to outcome devaluation observed in the present study. However, it has been shown that whatever the amount of instrumental training, stimulus-response habits do not overcome goal-directed decision making when two responses associated with different outcomes are concurrently available (Colwill and Rescorla, 1985; Holland, 2004; Kosaki and Dickinson, 2010). Alternatively, numerous studies have shown that cocaine exposure promotes habitual responding, whether for cocaine itself (Dickinson et al., 2002; Miles et al., 2003) or a nondrug reward (Gourley et al., 2013; LeBlanc et al., 2013; Corbit et al., 2014; Schmitzer-Torbert et al., 2015). However prior cocaine exposure was not sufficient to bias responding toward habit in a choice situation involving two nondrug rewards close in value, and cannot account for the results reported here (Halbout et al., 2016). Rather, the rapid development of habit may have been promoted by prior training in the discrete trial choice procedure. Indeed, it was shown that the insertion and retraction of the lever at the onset and termination of discrete trials constitute salient reward-predictive cues, leading to higher automaticity, behavioral chunking, and the rapid development of stimulus-bound habitual responding (Vandaele et al., 2017, 2019a).

An alternative explanation for the unexpected habitual performance in our choice procedure could reside in the large difference in saccharin and cocaine incentive value (Cantin et al., 2010). Theoretical models and a growing body of evidence suggest that the brain chooses advantageously among competing options by assigning values to the two stimuli, comparing them, and selecting the best course of action (Glimcher and Rustichini, 2004; Rangel et al., 2008; Rushworth et al., 2009; Rangel and Hare, 2010). Therefore, when the available options are difficult to distinguish, decisions are made based on careful evaluation of options values, and therefore, remain under goal-directed control. Consistent with this, choice performance is systematically under goal-directed control when the choice outcomes are close in value (Colwill and Triola, 2002; Holland, 2004; Kosaki and Dickinson, 2010). However, in our situation, the value difference between the two outcomes is such that decision-making does not require effortful representation and comparison of the value of the option and could instead rely on a simpler stimulus-response policy, based on prior reward history. As such, we suggest that the difference in options’ values might encourage the transition from goal-directed to habitual performance. Consistent with this hypothesis, Daw et al. (2005) suggested that arbitration between goal-directed and habitual systems relies on the relative uncertainty of predictions from each system with a low task complexity favoring habitual model-free control (Daw et al., 2005). Also, Keramati et al. (2011) proposed a normative model in which the relative incentive value of each outcome critically affects the arbitration between goal-directed and habit processes (Keramati et al., 2011). If the arbitration between goal-directed and habitual processes depends on the value difference between options, then one would expect that behavior would be goal-directed during a choice between cocaine and another nondrug reward with a similar reinforcing value, such as a lower concentration of saccharin (Cantin et al., 2010). This hypothesis remains to be tested in future experiments.

At a neurobiological level, the balance between goal-directed and habitual behavior depends upon corticostriatal circuits, with a sensorimotor–dorsolateral striatal network supporting habitual, stimulus-response behaviors and a prefrontal–dorsomedial striatal network mediating flexible, goal-directed behavior (Yin and Knowlton, 2004; Yin et al., 2005, 2006; Hitchcott et al., 2007; Ashby et al., 2010; Balleine and O’Doherty, 2010; Corbit et al., 2012; Lingawi and Balleine, 2012). Among regions of the “goal-directed network,” the orbitofrontal cortex (OFC) is critical when values must be used to guide responding based on a representation of the expected outcomes (Rushworth et al., 2011; Schoenbaum et al., 2011; Padoa-Schioppa and Conen, 2017). Although this region would be expected to support choice performance, recent studies reported no effect of optogenetic inhibition of OFC on economic choice behavior (Gardner et al., 2017, 2018). To explain this surprising result, the authors suggested that economic choice in their task may not be entirely governed by model-based goal-directed behavior but could instead rely on habits, as is the case in the present study. We have recently found neuronal correlates of preference between cocaine and saccharin in the OFC, with the size of the cocaine-signaling neuronal assembly during sampling trials predicting preference for cocaine during choice trials (Guillem and Ahmed, 2018, 2019). Future experiments will move one step forward to investigate the causal involvement of this region in choice performance during discrete-trial choices between cocaine and saccharin. From the results reported here, we should expect no effect of OFC lesion, pharmacological inactivation, or optogenetic inhibition on choice performance.

Several theories suggest that drugs of abuse may contribute to compulsive drug use by promoting habitual drug-seeking, at the expense of alternative activities (Robbins and Everitt, 1999; Everitt and Robbins, 2005, 2016). Although the difficulty to devalue drug self-administered intravenously precludes any conclusion about the nature of cocaine-seeking (i.e., habitual or not), our results do not seem consistent with these theories. In our study, habitual responding for saccharin may bias preference toward saccharin choice. Indeed, by definition habitual responding for saccharin is automatically triggered by antecedent stimuli (for instance, the insertion of the lever) with short response latency. In contrast, if responding for cocaine is under goal-directed control, then the selection of this option would require a representation of the outcome value and would be associated with longer response latencies. Preference for saccharin could then be explained by a faster selection of the saccharin option, as previously suggested (Shapiro et al., 2008). Analysis of sampling latencies supports this hypothesis with shorter sampling latency for saccharin compared to cocaine. Does this mean that preference for saccharin only results from habit? Previous findings suggest that habitual responding for saccharin is not sufficient to explain the preference (Vandaele et al., in press). Indeed, rats still preferred the saccharin option when they were able to exert voluntary goal-directed control over the initiation of choice trials. Furthermore, preference is sensitive to variation in saccharin concentration, delay, and cost (Lenoir et al., 2007; Cantin et al., 2010), suggesting that the value of saccharin is still computed and considered in the decision-making process, although with less deliberation than previously thought.

In conclusion, we report strong evidence that choice behavior can become habitual in a drug choice setting in rats. Prior training in the discrete trial choice procedure combined with the large difference in options value may have contributed to this finding. Clearly, more research is needed to understand why choice behavior between drug and nondrug rewards becomes habitual and inflexible in our conditions in comparison to other choice studies. This question is all the more important because growing evidence in humans suggests that habit formation occurs rarely, if at all, in similar laboratory drug choice settings (Hogarth, 2020). One major difference between these two sets of choice studies, in addition to species-specific differences, is that in human drug choice studies, people preferred the drug option over the nondrug option while in our and other studies, rats showed the opposite preference. Understanding this difference in drug preference may represent a first step toward understanding differential engagement of goal-directed control during drug choice in these two animal species.

## Data Availability Statement

The raw data supporting the conclusions of this article will be made available by the authors, without undue reservation.

## Ethics Statement

The animal studies were reviewed and approved by the Committee of the Veterinary Services Gironde, agreement number B33-063-5.

## Author Contributions

SA conceived the project. KG and SA designed the experiment. KG carried out the experiment and collected the data. KG and YV analyzed the data and wrote the first version of the manuscript. All authors critically edited, reviewed content and approved the final version for publication.

## Conflict of Interest

The authors declare that the research was conducted in the absence of any commercial or financial relationships that could be construed as a potential conflict of interest.

## References

[B1] Adams C. D. (1982). Variations in the sensitivity of instrumental responding to reinforcer devaluation. Q. J. Exp. Psychol. Sect. B 34, 77–98. 10.1080/14640748208400878

[B2] Ashby F. G. Turner B. O. Horvitz J. C. (2010). Cortical and basal ganglia contributions to habit learning and automaticity. Trends Cogn. Sci. 14, 208–215. 10.1016/j.tics.2010.02.001 20207189 PMC2862890

[B3] Augier E. Vouillac C. Ahmed S. H. (2012). Diazepam promotes choice of abstinence in cocaine self-administering rats. Addict. Biol. 17, 378–391. 10.1111/j.1369-1600.2011.00368.x 21955224

[B4] Balleine B. W. Dickinson A. (1998). Goal-directed instrumental action: contingency and incentive learning and their cortical substrates. Neuropharmacology 37, 407–419. 10.1016/s0028-3908(98)00033-1 9704982

[B5] Balleine B. W. O’Doherty J. P. (2010). Human and rodent homologies in action control: corticostriatal determinants of goal-directed and habitual action. Neuropsychopharmacology 35, 48–69. 10.1038/npp.2009.131 19776734 PMC3055420

[B6] Boakes R. A. Westbrook R. F. Elliott M. Swinbourne A. L. (1997). Context dependency of conditioned aversions to water and sweet tastes. J. Exp. Psychol. Anim. Behav. Process. 23, 56–67. 10.1037/0097-7403.23.1.56 9008862

[B7] Cantin L. Lenoir M. Augier E. Vanhille N. Dubreucq S. Serre F.. (2010). Cocaine is low on the value ladder of rats: possible evidence for resilience to addiction. PLoS One 5:e11592. 10.1371/journal.pone.0011592 20676364 PMC2911372

[B8] Colwill R. M. Rescorla R. A. (1985). Instrumental responding remains sensitive to reinforcer devaluation after extensive training. J. Exp. Psychol. Anim. Behav. Process. 11, 520–536. 10.1037/0097-7403.11.4.520

[B9] Colwill R. M. Triola S. M. (2002). Instrumental responding remains under the control of the consequent outcome after extended training. Behav. Processes 57, 51–64. 10.1016/s0376-6357(01)00204-2 11864775

[B10] Corbit L. H. Chieng B. C. Balleine B. W. (2014). Effects of repeated cocaine exposure on habit learning and reversal by N-acetylcysteine. Neuropsychopharmacology 39, 1893–1901. 10.1038/npp.2014.37 24531561 PMC4059898

[B11] Corbit L. H. Leung B. K. Balleine B. W. (2013). The role of the amygdala-striatal pathway in the acquisition and performance of goal-directed instrumental actions. J. Neurosci. 33, 17682–17690. 10.1523/JNEUROSCI.3271-13.2013 24198361 PMC6618427

[B12] Corbit L. Nie H. Janak P. (2012). Habitual alcohol seeking: time course and the contribution of subregions of the dorsal striatum. Biol. Psychiatry 72, 389–395. 10.1016/j.biopsych.2012.02.024 22440617 PMC3674580

[B13] Coutureau E. Killcross S. (2003). Inactivation of the infralimbic prefrontal cortex reinstates goal-directed responding in overtrained rats. Behav. Brain Res. 146, 167–174. 10.1016/j.bbr.2003.09.025 14643469

[B14] Daw N. D. Niv Y. Dayan P. (2005). Uncertainty-based competition between prefrontal and dorsolateral striatal systems for behavioral control. Nat. Neurosci. 8, 1704–1711. 10.1038/nn1560 16286932

[B15] Dickinson A. (1985). Actions and habits: the development of behavioural autonomy. Philos. Trans. R. Soc. B Biol. Sci. 308, 67–78. 10.1098/rstb.1985.0010

[B16] Dickinson A. (1994). “Instrumental conditioning,” in Handbook of Perception and Cognition Series, ed. Mackintosh N. J. (San Diego CA: Academic Press), 45–79.

[B17] Dickinson A. Balleine B. (1994). Motivational control of instrumental action. Anim. Learn. Behav. 22, 1–18. 10.3758/BF03199951

[B18] Dickinson A. Balleine B. W. Watt A. Gonzalez F. Boakes R. A. (1995). Motivational control after extended instrumental training. Anim. Learn. Behav. 23, 197–206. 10.3758/bf03199935

[B4900] Dickinson A. Nicholas D. J. Adams C. D. (1983). The effect of the instrumental training contingency on susceptibility to reinforcer devaluation. Q. J. Exp. Psychol. Sect. B 35, 35–51. 10.1080/1464074830840091219349160

[B19] Dickinson A. Wood N. Smith J. W. (2002). Alcohol seeking by rats: action or habit? Q. J. Exp. Psychol. B 55, 331–348. 10.1080/0272499024400016 12350285

[B20] Everitt B. J. Robbins T. W. (2005). Neural systems of reinforcement for drug addiction: from actions to habits to compulsion. Nat. Neurosci. 8, 1481–1489. 10.1038/nn1579 16251991

[B21] Everitt B. J. Robbins T. W. (2016). Drug addiction: updating actions to habits to compulsions ten years on. Annu. Rev. Psychol. 67, 23–50. 10.1146/annurev-psych-122414-033457 26253543

[B23] Gardner M. P. H. Conroy J. S. Shaham M. H. Styer C. V. Schoenbaum G. (2017). Lateral orbitofrontal inactivation dissociates devaluation-sensitive behavior and economic choice. Neuron 96, 1192.e4–1203.e4. 10.1016/j.neuron.2017.10.026 29154127 PMC5728681

[B22] Gardner M. P. H. Conroy J. C. Styer C. V. Huynh T. Whitaker L. R. Schoenbaum G. (2018). Medial orbitofrontal inactivation does not affect economic choice. Elife 7:e38963. 10.7554/eLife.38963 30281020 PMC6170187

[B24] Glimcher P. W. Rustichini A. (2004). Neuroeconomics: the consilience of brain and decision. Science 306, 447–452. 10.1126/science.1102566 15486291

[B25] Gourley S. L. Olevska A. Gordon J. Taylor J. R. (2013). Cytoskeletal determinants of stimulus-response habits. J. Neurosci. 33, 11811–11816. 10.1523/JNEUROSCI.1034-13.2013 23864670 PMC3713723

[B26] Guillem K. Ahmed S. H. (2018). Preference for cocaine is represented in the orbitofrontal cortex by an increased proportion of cocaine use-coding neurons. Cereb. Cortex 28, 819–832. 10.1093/cercor/bhw398 28057724

[B27] Guillem K. Ahmed S. H. (2019). A neuronal population code for resemblance between drug and nondrug reward outcomes in the orbitofrontal cortex. Brain Struct. Funct. 224, 883–890. 10.1007/s00429-018-1809-8 30539287

[B28] Halbout B. Liu A. T. Ostlund S. B. (2016). A closer look at the effects of repeated cocaine exposure on adaptive decision-making under conditions that promote goal-directed control. Front. Psychiatry 7:44. 10.3389/fpsyt.2016.00044 27047400 PMC4800177

[B29] Hart G. Leung B. K. Balleine B. W. (2014). Dorsal and ventral streams: the distinct role of striatal subregions in the acquisition and performance of goal-directed actions. Neurobiol. Learn. Mem. 108, 104–118. 10.1016/j.nlm.2013.11.003 24231424 PMC4661143

[B30] Hitchcott P. K. Quinn J. J. Taylor J. R. (2007). Bidirectional modulation of goal-directed actions by prefrontal cortical dopamine. Cereb. Cortex 17, 2820–2827. 10.1093/cercor/bhm010 17322558

[B31] Hogarth L. (2020). Addiction is driven by excessive goal-directed drug choice under negative affect: translational critique of habit and compulsion theory. Neuropsychopharmacology 45, 720–735. 10.1038/s41386-020-0600-8 31905368 PMC7265389

[B32] Holland P. C. (2004). Relations between Pavlovian-instrumental transfer and reinforcer devaluation. J. Exp. Psychol. Anim. Behav. Process. 30, 104–117. 10.1037/0097-7403.30.2.104 15078120

[B510] Keiflin R. Pribut H. J. Shah N. B. Janak P. H. (2019). Ventral tegmental dopamine neurons participate in reward identity predictions. Curr. Biol. 29, 93–103. 10.1016/j.cub.2018.11.050 30581025 PMC6324975

[B33] Keramati M. Dezfouli A. Piray P. (2011). Speed/accuracy trade-off between the habitual and the goal-directed processes. PLoS Comput. Biol. 7:e1002055. 10.1371/journal.pcbi.1002055 21637741 PMC3102758

[B34] Kislal S. Blizard D. A. (2016). Conditioned context aversion learning in the laboratory mouse. Learn. Behav. 44, 309–319. 10.3758/s13420-016-0217-2 26961783

[B35] Kislal S. Blizard D. A. (2018). Acquisition and retention of conditioned aversions to context and taste in laboratory mice. Learn. Behav. 46, 198–212. 10.3758/s13420-017-0303-0 29124570

[B36] Kosaki Y. Dickinson A. (2010). Choice and contingency in the development of behavioral autonomy during instrumental conditioning. J. Exp. Psychol. Anim. Behav. Process. 36, 334–342. 10.1037/a0016887 20658864

[B37] LeBlanc K. H. Maidment N. T. Ostlund S. B. (2013). Repeated cocaine exposure facilitates the expression of incentive motivation and induces habitual control in rats. PLoS One 8:e61355. 10.1371/journal.pone.0061355 23646106 PMC3640016

[B38] Lenoir M. Augier E. Vouillac C. Ahmed S. H. (2013). A Choice-based screening method for compulsive drug users in rats. Curr. Protoc. Neurosci. 64, 9.44.1–9.44.17. 10.1002/0471142301.ns0944s64 23853111

[B39] Lenoir M. Serre F. Cantin L. Ahmed S. H. (2007). Intense sweetness surpasses cocaine reward. PLoS One 2:e698. 10.1371/journal.pone.0000698 17668074 PMC1931610

[B40] Lingawi N. W. Balleine B. W. (2012). Amygdala central nucleus interacts with dorsolateral striatum to regulate the acquisition of habits. J. Neurosci. 32, 1073–1081. 10.1523/JNEUROSCI.4806-11.2012 22262905 PMC3711777

[B41] Madsen H. B. Ahmed S. H. (2015). Drug versus sweet reward: greater attraction to and preference for sweet versus drug cues. Addict. Biol. 20, 433–444. 10.1111/adb.12134 24602027

[B42] Miles F. J. Everitt B. J. Dickinson A. (2003). Oral cocaine seeking by rats: action or habit? Behav. Neurosci. 117, 927–938. 10.1037/0735-7044.117.5.927 14570543

[B43] Padoa-Schioppa C. Conen K. E. (2017). Orbitofrontal cortex: a neural circuit for economic decisions. Neuron 96, 736–754. 10.1016/j.neuron.2017.09.031 29144973 PMC5726577

[B44] Parkes S. L. Balleine B. W. (2013). Incentive memory: evidence the basolateral amygdala encodes and the insular cortex retrieves outcome values to guide choice between goal-directed actions. J. Neurosci. 33, 8753–8763. 10.1523/JNEUROSCI.5071-12.2013 23678118 PMC3717368

[B46] Rangel A. Camerer C. Montague P. R. (2008). A framework for studying the neurobiology of value-based decision making. Nat. Rev. Neurosci. 9, 545–556. 10.1038/nrn2357 18545266 PMC4332708

[B45] Rangel A. Hare T. (2010). Neural computations associated with goal-directed choice. Curr. Opin. Neurobiol. 20, 262–270. 10.1016/j.conb.2010.03.001 20338744

[B47] Robbins T. W. Everitt B. J. (1999). Drug addiction: bad habits add up. Nature 398, 567–570. 10.1038/19208 10217139

[B49] Rushworth M. F. Mars R. B. Summerfield C. (2009). General mechanisms for making decisions? Curr. Opin. Neurobiol. 19, 75–83. 10.1016/j.conb.2009.02.005 19349160

[B48] Rushworth M. F. S. Noonan M. A. P. Boorman E. D. Walton M. E. Behrens T. E. (2011). Frontal cortex and reward-guided learning and decision-making. Neuron 70, 1054–1069. 10.1016/j.neuron.2011.05.014 21689594

[B50] Schmitzer-Torbert N. Apostolidis S. Amoa R. O’Rear C. Kaster M. Stowers J.. (2015). Post-training cocaine administration facilitates habit learning and requires the infralimbic cortex and dorsolateral striatum. Neurobiol. Learn. Mem. 118, 105–112. 10.1016/j.nlm.2014.11.007 25460040 PMC4331257

[B470] Schoenbaum G. Setlow B. (2005). Cocaine makes actions insensitive to outcomes but not extinction: implications for altered orbitofrontal-amygdalar function. Cereb. Cortex 15, 1162–1169. 10.1093/cercor/bhh216 15563719

[B51] Schoenbaum G. Takahashi Y. Liu T. L. McDannald M. A. (2011). Does the orbitofrontal cortex signal value? Ann. N Y Acad. Sci. 1239, 87–99. 10.1111/j.1749-6632.2011.06210.x 22145878 PMC3530400

[B52] Shapiro M. S. Siller S. Kacelnik A. (2008). Simultaneous and sequential choice as a function of reward delay and magnitude: normative, descriptive and process-based models tested in the european starling (Sturnus vulgaris). J. Exp. Psychol. Anim. Behav. Process. 34, 75–93. 10.1037/0097-7403.34.1.75 18248116

[B53] Vandaele Y. Cantin L. Serre F. Vouillac-Mendoza C. Ahmed S. H. (2016). Choosing under the influence: a drug-specific mechanism by which the setting controls drug choices in rats. Neuropsychopharmacology 41, 646–657. 10.1038/npp.2015.195 26129679 PMC5130140

[B54] Vandaele Y. Mahajan N. R. Ottenheimer D. J. Richard J. M. Mysore S. P. Janak P. H. (2019a). Distinct recruitment of dorsomedial and dorsolateral striatum erodes with extended training. Elife 8:e49536. 10.7554/eLife.49536 31621583 PMC6822989

[B56] Vandaele Y. Vouillac-Mendoza C. Ahmed S. H. (2019b). Inflexible habitual decision-making during choice between cocaine and a nondrug alternative. Transl. Psychiatry 9:109. 10.1038/s41398-019-0445-2 30842406 PMC6403316

[B57] Vandaele Y. Vouillac-Mendoza C. Ahmed S. H. (in press). Cocaine falls into oblivion during volitional initiation of choice trials. Addict. Biol. 10.1111/adb.13235 36301214

[B55] Vandaele Y. Pribut H. J. Janak P. H. (2017). Lever insertion as a salient stimulus promoting insensitivity to outcome devaluation. Front. Integr. Neurosci. 11:23. 10.3389/fnint.2017.00023 29021746 PMC5623688

[B58] Yin H. H. Knowlton B. J. (2004). Contributions of striatal subregions to place and response learning. Learn. Mem. 11, 459–463. 10.1101/lm.81004 15286184 PMC498333

[B59] Yin H. H. Knowlton B. J. (2006). The role of the basal ganglia in habit formation. Nat. Rev. Neurosci. 7, 464–476. 10.1038/nrn1919 16715055

[B60] Yin H. H. Knowlton B. J. Balleine B. W. (2006). Inactivation of dorsolateral striatum enhances sensitivity to changes in the action-outcome contingency in instrumental conditioning. Behav. Brain Res. 166, 189–196. 10.1016/j.bbr.2005.07.012 16153716

[B61] Yin H. H. Ostlund S. B. Knowlton B. J. Balleine B. W. (2005). The role of the dorsomedial striatum in instrumental conditioning. Eur. J. Neurosci. 22, 513–523. 10.1111/j.1460-9568.2005.04218.x 16045504

